# Single Nucleotide Polymorphisms in the Human Leukocyte Antigen Region Are Associated With Hemagglutination Inhibition Antibody Response to Influenza Vaccine

**DOI:** 10.3389/fgene.2022.790914

**Published:** 2022-02-07

**Authors:** Shuyi Zhong, Hejiang Wei, Mao Li, Yanhui Cheng, Simin Wen, Dayan Wang, Yuelong Shu

**Affiliations:** ^1^ School of Public Health (Shenzhen), Shenzhen Campus of Sun Yat-sen University, Shenzhen, China; ^2^ Institute for Viral Disease Control and Prevention, Chinese Center for Disease Prevention and Control, Beijing, China

**Keywords:** influenza vaccine, hemagglutination inhibition antibody, human leukocyte antigen, single nucleotide polymorphisms, genetic epidemiology

## Abstract

**Background:** The annual death associated with seasonal influenza is 290,000–650,000 globally, which can be effectively reduced by influenza vaccination. However, the protective hemagglutination inhibition (HAI) antibody response to influenza vaccine is affected by many factors, among which single nucleotide polymorphisms (SNPs) in the human leukocyte antigen (HLA) region can alter the antigen-presenting function of the HLA molecule, thus influencing the process of antibody mounting against vaccine antigen.

**Methods:** Healthy subjects of the Han nationality were recruited and received seasonal trivalent influenza vaccine. Paired serum samples collected on and approximately 28 days after vaccination were tested in parallel by HAI assays. HLA alleles related to the immune response to influenza vaccine reported in the previous literature were summarized, and six corresponding tag SNPs were selected and genotyped using the MassARRAY technology platform.

**Results:** The effects of HLA SNPs on HAI antibody response to influenza vaccine varied with different vaccine antigens. The AA genotype of rs41547618 was correlated with low A/H1N1-specific antibody titer compared with the GG + GA genotype (*p* = .007). The TT genotype of rs17885382 was correlated with low A/H3N2-specific antibody titer compared with the CC + CT genotype (*p* = .003). In addition, haplotype consisting of rs41542812—rs17885382—rs2068205—rs41547618—rs6905837—rs9270299—CCTGCA was correlated with non-responsiveness to influenza vaccine (OR = 2.39, 95% CI = 1.02–5.62).

**Conclusion:** HLA SNPs were associated with HAI antibody response to influenza vaccine, which can help in a better understanding of the varied responsiveness to influenza vaccine in the population.

## 1 Introduction

Influenza remains one of the main threats to public health worldwide, resulting in seasonal epidemics with 290–650 thousand deaths annually (Iuliano et al., 2018). Vaccination was proved to be one of the most effective measurements to reduce the infection risk of influenza virus by 40%–60% in the overall population during the season when the vaccine strains well-matched the circulating ones (Centers for Disease Control and Prevention, 2021). However, influenza vaccine is not universally protective, and there are still some individuals who fail to mount a protective serologic response to the vaccine. The immune response to influenza vaccine may be influenced by host-related factors such as age, gender, health condition, and genetic variants (Powers and Belshe, 1993; Castrucci, 2018; Scepanovic et al., 2018).

A few studies have found that genetic variants in human leukocyte antigen (HLA), cytokine, and cytokine receptors may partly explain the variability of individual vaccine responsiveness (Poland et al., 2007; Posteraro et al., 2014; Linnik and Egli, 2016). The HLA gene encodes regulatory elements involved in adaptive immunity, and it is also one of the most polymorphic regions in the human genome. Single nucleotide polymorphisms (SNPs) may change the binding region of HLA molecular peptides, thus affecting its role in the presentation of the influenza vaccine antigen. Several HLA allelic variants were found to be significantly associated with response to influenza vaccine (Gelder et al., 2002; Lambkin et al., 2004; Poland et al., 2008; Moss et al., 2013; Narwaney et al., 2013). However, given the abundance of HLA alleles and multiple research designs, the results of the relevant literature are somewhat sporadic.

To further explore the effects of the HLA gene on immune response to influenza vaccine, we selected six SNPs in the HLA region based on related reports and analyzed their association with hemagglutination inhibition (HAI) antibody response to influenza vaccine, which may also provide a better understanding of the variability of immune response to influenza vaccine.

## 2 Materials and Methods

### 2.1 Study Population and Vaccinations

**FIGURE 1:**
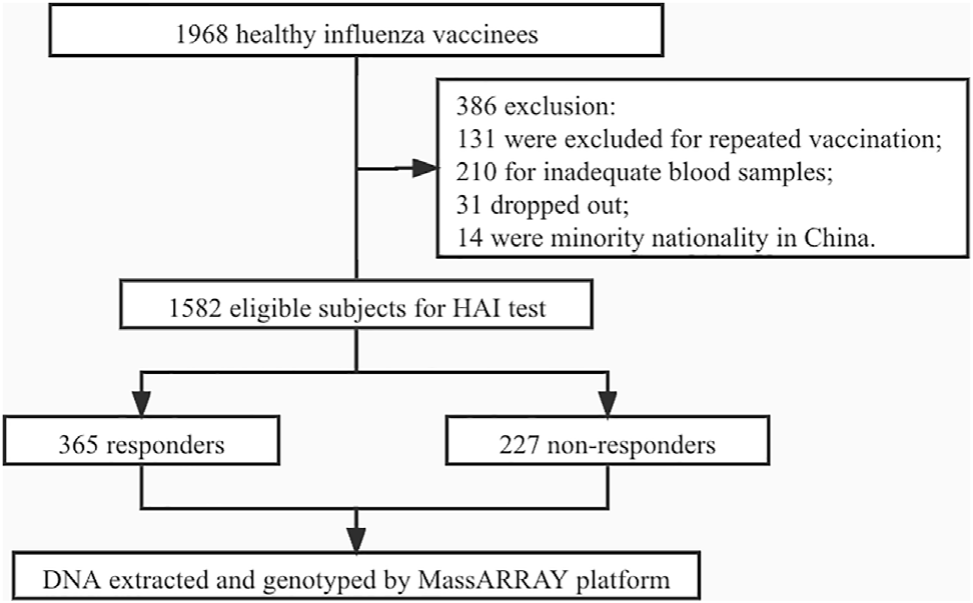
Flow chart.

From 2009 to 2019, 1968 healthy subjects were recruited from the Yunnan Center for Disease Control and Prevention (CDC) and the Urumqi CDC with informed consent. The subjects were excluded from the enrollment if they were non-Han nationality or with a history of prior influenza vaccination. As a result, 1582 subjects were eligible for further study, and 386 subjects were excluded with reasons, which were described in Figure 1. Study procedures were approved by the Ethics Review Committee of National Institute for Viral Disease Control and Prevention, Chinese CDC (reference no. 200916, 2010).

All participants received trivalent inactivated influenza vaccine by intramuscular injections. Influenza vaccines that were used for adults contained 15 µg hemagglutination (HA) for each strain and were composed as recommended by the World Health Organization (WHO) for the northern hemisphere, details of which can be found in Supplementary Table S1. Children under 5 years old received two doses of influenza vaccine with an interval of 30 days. The blood samples were obtained on the vaccination day and approximately 28 days after the accomplishment of full vaccination. The paired serum samples were isolated immediately and stored at −80°C before tests.

### 2.2 Hemagglutination Inhibition Assay

The paired serum samples were tested in parallel by HAI assays using 1% red blood cells from turkey or guinea pigs to measure the endpoint of HAI antibody titers against all vaccine strains. According to the standard reagent preparation protocols from WHO, influenza virus antigens were cultured by specified pathogen-free (SPF) chicken embryo. Serum antibody titers were ascertained as the highest fold of dilution which could completely lead to HAI, and 60 µl receptor-destroying enzyme (RDE, Denka Seiken, Japan) was added to 20 µl serum to remove the non-specific inhibitors in 37°C water-bath for 16–18 h. Then, the compound was inactivated by 56°C water bath for 30 min before adding 20 µl phosphate buffered saline (PBS, Biosharp, China). The serums were tested in two-fold serial dilutions with an initial fold of 1:5.

The seroconversion rate was defined as the percentage of participants mounting to at least 1:40 in the postvaccination titer from the pre-vaccination titer less than 1:10 or achieving at least four-fold increase in the postvaccination titer to the pre-vaccination antibody titer. Responder was defined as those who achieved seroconversion to all vaccine composition strains, and non-responder was those who did not achieve seroconversion to all vaccine composition strains. According to the definitions, 365 responders and 227 non-responders were selected for genotyping.

### 2.3 SNP Selection and Genotyping

We systematically summarized the HLA alleles related to the immune response to influenza vaccine reported in the previous literature. Then, we selected the corresponding tag SNPs of those alleles based on the HLA gene sequencing database of 20635 Chinese Han people (Zhou et al., 2016). Among them, six SNPs with the minimum allele frequencies (MAFs) greater than 0.05 in the Han nationality were finally chosen in this study. The relationship between SNPs and their corresponding HLA alleles and detection rates are shown in Supplementary Table S2.

The DNA was extracted from the blood using the TIANamp Blood Clot DNA Kit (TIANGEN, DP201101X) according to the manufacturer’s protocols. Genotyping of SNP was performed using the MassARRAY technology platform (Sequenom, San Diego, California, United States) by BioMiao Biological Technology (Beijing, China).

### 2.4 Statistical Analysis

Data cleaning and statistical analysis were performed using SPSS version 26. Chi-squared goodness-of-fit was used to test deviations of the SNP genotype frequency from the Hardy–Weinberg equilibrium. Distributions of genotype frequency between responder and non-responder groups were tested using the chi-square test or Fisher’s exact test. Logistic regression models were used to estimate the effect of SNPs in the HLA gene on the responsiveness to influenza vaccine adjusted for age and gender. To fit the normality assumptions, HAI titers were transformed to their log values (base 10) before analysis.

The relationship between the HLA SNP genotype and influenza vaccine antibody titer was analyzed by covariance analysis after adjusting for gender, age, regions, and preexisting (Day 0) antibody titer. The linkage disequilibrium (LD) test and haplotype analysis were performed on the website SNPStats (https://www.snpstats.net).

Statistical significance was defined as *p* < .05 or 95% confidence interval (CI) excluding 1. In the analysis of the relationship between SNPs and HAI antibody response to influenza vaccine, the significance level should be turned to *p* < .008 (.05/6 = .008) according to the Bonferroni correction.

## 3 Results

### 3.1 Characteristics of Subjects

A total of 592 subjects were included in the final analysis. As shown in Table 1, there are 227 non-responders and 365 responders. There were no significant differences in gender, age, and regions between the two groups.

**TABLE 1:** Demographic characteristics and HAI antibody titers of the study subjects.

Variable	Overall (*N* = 592)	Non-responder (*N* = 227)	Responder (*N* = 365)	*p*
Gender (n, %)				
Male	233 (39.4)	91 (40.1)	142 (38.9)	.774^a^
Female	359 (60.6)	136 (59.9)	223 (61.1)	
Age group (n, %)				
<5	138 (23.3)	54 (23.8)	84 (23.0)	.102^a^
5∼17	61 (10.3)	16 (7.1)	45 (12.3)	
18∼64	283 (47.8)	107 (47.1)	176 (48.3)	
≥65	110 (18.6)	50 (22.0)	60 (16.4)	
Regions (n, %)				
Xinjiang	334 (56.4)	137 (60.4)	197 (54.0)	.128^a^
Yunnan	258 (43.6)	90 (39.6)	168 (46.0)	
HAI titer (median, IQR)				
Day 0				
A/H1N1	1:10 (1:5–1:40)	1:20 (1:5–1:80)	1:5 (1:5–1:20)	<.001^b^
A/H3N2	1:20 (1:10–1:40)	1:20 (1:10–1:80)	1:20 (1:10–1:40)	.081^b^
B/Victoria	1:10 (1:5–1:20)	1:10 (1:5–1:20)	1:10 (1:5–1:20)	.055^b^
B/Yamagata	1:20 (1:5–1:80)	1:60 (1:10–1:320)	1:10 (1:5–1:40)	<.001^b^
Day 28				
A/H1N1	1:80 (1:20–1:320)	1:20 (1:10–1:80)	1:320 (1:80–1:640)	<.001^b^
A/H3N2	1:160 (1:40–1:320)	1:20 (1:10–1:80)	1:320 (1:80–1:640)	<.001^b^
B/Victoria	1:80 (1:40–1:320)	1:160 (1:80–1:320)	1:160 (1:80–1:320)	<.001^b^
B/Yamagata	1:160 (1:40–1:640)	1:40 (1:12.5–1:320)	1:320 (1:80–1:1280)	<.001^b^

a Calculated by the chi-square test.

b Calculated by the Mann–Whitney U test.

The preexisting antibody was demonstrated by the baseline (Day 0) strain-specific HAI antibody titer. Comparing the median HAI titer values between the two groups, the responders had a significantly lower baseline titer to A/H1N1 and B/Yamagata than non-responders (*p* < .001). On day 28, after vaccination, specific antibody titers of all strains were significantly lower in non-responders than those in responders (*p* < .001).

### 3.2 Association Between SNPs and Responsiveness to Influenza Vaccine

All genotype frequencies were in the Hardy–Weinberg equilibrium with call rates higher than 95%. The results in Table 2 show no significant difference in SNP frequency between responder and non-responder groups.

**TABLE 2:** HLA SNPs genotype frequencies in responder and non-responder groups.

SNP	Genotype	Non-responder N (%)	Responder N (%)	*P* ^a^	OR (95%CI)^b^	*P* ^b^
rs41542812	CC	194 (86.6)	298 (82.8)	0.363	1.00	
	GC	16 (7.1)	28 (7.8)		1.14 (.60–2.16)	.697
	GG	14 (6.2)	34 (9.4)		1.60 (.83–3.06)	.159
	C	404 (90.2)	624 (86.7)	.088		
	G	44 (9.8)	96 (13.3)			
rs17885382	CC	173 (81.2)	297 (84.1)	.580	1.00	
	CT	15 (7.0)	24 (6.8)		.96 (0.49–1.88)	.894
	TT	25 (11.7)	32 (9.1)		.76 (0.43–1.32)	0.327
	C	361 (84.7)	618 (87.5)	.214		
	T	65 (15.3)	88 (12.5)			
rs2068205	CC	167 (74.6)	285 (79.2)	.388	1.00	
	CT	52 (23.2)	70 (19.4)		.78 (.52–1.17)	0.226
	TT	5 (2.2)	5 (1.4)		0.58 (0.16–2.03)	0.392
	C	386 (86.2)	640 (88.9)	0.195		
	T	62 (13.8)	80 (11.1)			
rs41547618	GG	132 (59.2)	212 (59.2)	0.428	1.00	
	GA	67 (30.0)	118 (33.0)		1.07 (0.74–1.56)	0.714
	AA	24 (10.8)	28 (7.8)		0.71 (0.39–1.28)	0.257
	G	331 (74.2)	542 (75.7)	0.618		
	A	115 (25.8)	174 (24.3)			
rs6905837	CC	158 (71.2)	267 (76.)	0.290	1.00	
	CT	57 (25.7)	70 (20.1)		0.73 (0.49–1.09)	0.125
	TT	7 (3.2)	12 (3.4)		1.05 (0.40–2.75)	0.916
	C	373 (84.0)	604 (86.5)	0.273		
	T	71 (16.0)	94 (13.5)			
rs9270299	CC	160 (73.4)	260 (74.3)	0.293	1.00	
	CA	16 (7.3)	36 (10.3)		1.46 (0.78–2.73)	0.236
	AA	42 (19.3)	54 (15.4)		0.78 (0.50–1.22)	0.277
	C	336 (77.1)	556 (79.4)	0.385		
	A	100 (22.9)	144 (20.6)			

a Calculated by the chi-square test.

b Adjusted for age and gender by logistic regression models.

Association between SNPs and strain-specific antibody titers to influenza vaccine.

The results in Table 3 show that two SNPs (rs41547618 and rs41542812) correlated with variations of A/H1N1-specific antibody titers. The A/H1N1-specific antibody titers were not all the same among the three genotype carriers of rs41547618 (*p* = .006), and AA genotype correlated with low A/H1N1-specific antibody titer compared with the GG + GA genotype (*p* = .007), which was still significant after the Bonferroni correction. The GG genotype of rs41542812 correlated with a high A/H1N1-specific antibody titer compared with the CC + CG genotype (*p* = .039), but the difference was not significant after the Bonferroni correction.

**TABLE 3:** Results of covariance analysis for A/H1N1 antibody titers.

SNP	Model	Genotype	N (%)	mean ± SD	*P* ^a^
rs41542812	Additive	CC	492 (84.2)	1.99 ± .72	.085
		CG	44 (7.5)	1.90 ± .75	
		GG	48 (8.2)	2.20 ± .67	
	Recessive	CC + CG	536 (91.8)	1.99 ± .72	.039
		GG	48 (8.2)	2.20 ± .67	
rs17885382	Additive	CC	470 (83.0)	2.02 ± .71	.187
		CT	39 (6.9)	2.13 ± .77	
		TT	57 (10.1)	1.87 ± .79	
rs2068205	Additive	CC	452 (77.4)	2.01 ± .70	.605
		CT	122 (20.9)	2.00 ± .76	
		TT	10 (1.7)	1.78 ± .97	
rs41547618	Additive	GG	344 (59.2)	1.99 ± .72	**.006**
		GA	185 (31.8)	2.10 ± .73	
		AA	52 (9.0)	1.75 ± .63	
	Recessive	GG + GA	529 (91.0)	2.03 ± .73	**.007**
		AA	52 (9.0)	1.75 ± .63	
rs6905837	Additive	CC	425 (74.4)	2.00 ± .72	.975
		CT	127 (22.2)	2.00 ± .71	
		TT	19 (3.3)	1.97 ± .84	
rs9270299	Additive	CC	420 (73.9)	1.97 ± .72	.207
		CA	52 (9.2)	2.14 ± .78	
		AA	96 (16.9)	2.05 ± .68	

a Adjusted for gender, age, regions, and preexisting (Day 0) antibody titer.

Bold *p* values are significant after the Bonferroni correction.

As shown in Tables 3, 4, SNPs (rs17885382, rs2068205, and rs6905837) were associated with variations of A/H3N2-specific antibody titers. The A/H3N2-specific antibody titers were not all the same among the three genotype carriers of rs17885382 (*p* = .012), and the TT genotype was associated with low A/H3N2-specific antibody titer compared with the CC + CT genotype (*p* = .003), which was still significant after the Bonferroni correction. The A/H3N2-specific antibody titers were not all the same among the three genotype carriers of rs2068205 (*p* = .024), and the CT + TT genotype was associated with low A/H3N2-specific antibody titer compared with the CC genotype (*p* = .010), though the difference was not significant after the Bonferroni correction. Besides, the CT genotype of rs6905837 was associated with low A/H3N2-specific antibody titer compared with the CC + TT genotype (*p* = .015), but the correlation was not statistically significant after the Bonferroni correction.

**TABLE 4:** Results of covariance analysis for A/H3N2 antibody titers.

SNP	Model	Genotype	N (%)	mean ± SD	*P* ^a^
rs41542812	Additive	CC	492 (84.2)	2.06 ± .70	.120
		CG	44 (7.5)	2.14 ± .72	
		GG	48 (8.2)	2.24 ± .71	
rs17885382	Additive	CC	470 (83.0)	2.12 ± .70	.012
		CT	39 (6.9)	2.18 ± .72	
		TT	57 (10.1)	1.87 ± .62	
	Recessive	CC + CT	509 (89.9)	2.13 ± .70	**.003**
		TT	57 (10.1)	1.87 ± .62	
rs2068205	Additive	CC	452 (77.4)	2.11 ± .69	.024
		CT	122 (20.9)	1.96 ± .70	
		TT	10 (1.7)	1.78 ± .81	
	Dominant	CC	452 (77.4)	2.11 ± .69	.010
		CT + TT	132 (22.6)	1.95 ± .71	
rs41547618	Additive	GG	344 (59.2)	2.04 ± .72	.300
		GA	185 (31.8)	2.13 ± .69	
		AA	52 (9.0)	2.09 ± 0.67	
rs6905837	Additive	CC	425 (74.4)	2.11 ± .69	.050
		CT	127 (22.2)	1.96 ± .72	
		TT	19 (3.3)	2.14 ± .75	
	Overdominant	CC + TT	444 (77.8)	2.11 ± .69	.015
		CT	127 (22.2)	1.96 ± 0.72	
rs9270299	Additive	CC	420 (73.9)	2.07 ± .70	.331
		CA	52 (9.2)	2.20 ± .71	
		AA	96 (16.9)	2.07 ± .70	

a Adjusted for gender, age, regions, and preexisting (Day 0) antibody titer.

Bold *p* values are significant after the Bonferroni correction.

Our data found no SNPs associated with B/Victoria- or B/Yamagata-specific antibody titer after the Bonferroni correction (*p* > .008). The CC + CT genotype of rs17885382 was associated with high B/Victoria-specific antibody titer compared with the TT genotype (*p* = .036), although it was not significant after the Bonferroni correction. As for B/Yamagata, rs17885382, rs41547618, and rs6905837 were correlated with variation of the strain-specific antibody titer: the CC + CT genotype of rs17885382 was associated with high antibody titer compared to the TT genotype (*p* = .031), the GG + AA genotype of rs41547618 was associated with low antibody titer compared to the GA genotype (*p* = 0.024), and the CC + TT genotype of rs6905837 was associated with high antibody titer compared to the CT genotype (*p* = .043). However, the differences were not significant after the Bonferroni correction. The details are shown in Tables 5, 6.

**TABLE 5:** Results of covariance analysis for B/Victoria antibody titers.

SNP	Model	Genotype	N (%)	mean ± SD	*P* ^a^
rs41542812	Additive	CC	352 (84.4)	1.93 ± .71	.515
		CG	31 (7.4)	2.01 ± .67	
		GG	34 (8.2)	1.83 ± .59	
rs17885382	Additive	CC	328 (82.4)	1.96 ± .68	.032
		CT	29 (7.3)	2.15 ± .64	
		TT	41 (10.3)	1.76 ± .73	
	Recessive	CC + CT	357 (89.7)	1.98 ± .68	.036
		TT	41 (10.3)	1.76 ± .73	
rs2068205	Additive	CC	328 (78.5)	1.93 ± .68	.140
		CT	83 (19.9)	1.99 ± .75	
		TT	7 (1.7)	1.52 ± .96	
rs41547618	Additive	GG	249 (60.4)	1.91 ± .69	.612
		GA	132 (32.0)	1.97 ± .73	
		AA	31 (7.5)	1.86 ± .63	
rs6905837	Additive	CC	306 (75.4)	1.94 ± .69	.282
		CT	92 (22.7)	1.85 ± .71	
		TT	8 (2.0)	2.17 ± .76	
rs9270299	Additive	CC	306 (76.3)	1.90 ± .72	.627
		CA	34 (8.5)	1.99 ± .68	
		AA	61 (15.2)	1.96 ± .63	

a Adjusted for gender, age, regions, and preexisting (Day 0) antibody titer.

**TABLE 6:** Results of covariance analysis for B/Yamagata antibody titers.

SNP	Model	Genotype	N (%)	mean ± SD	*P* ^a^
rs41542812	Additive	CC	140 (83.8)	2.15 ± .73	.193
		CG	13 (7.8	1.97 ± .59	
		GG	14 (8.4)	2.42 ± .73	
rs17885382	Additive	CC	142 (84.5)	2.19 ± .75	.088
		CT	10 (6.0)	2.08 ± .52	
		TT	16 (9.5)	1.81 ± .60	
	Recessive	CC + CT	152 (90.5)	2.18 ± .74	.031
		TT	16 (9.5)	1.81 ± .60	
rs2068205	Additive	CC	124 (74.7)	2.16 ± .73	.912
		CT	39 (23.5)	2.14 ± 0.70	
		TT	3 (1.8)	2.00 ± 1.22	
rs41547618	Additive	GG	95 (56.2)	2.07 ± .72	.077
		GA	53 (31.4)	2.32 ± .72	
		AA	21 (12.4)	2.10 ± .76	
	Overdominant	GG + AA	116 (68.6)	2.08 ± .72	.024
		GA	53 (31.4)	2.32 ± .72	
rs6905837	Additive	CC	119 (72.1)	2.21 ± .71	.127
		CT	35 (21.2)	1.95 ± .82	
		TT	11 (6.7)	2.18 ± .65	
	Overdominant	CC + TT	130 (78.8)	2.21 ± .70	.043
		CT	35 (21.2)	1.95 ± .82	
rs9270299	Additive	CC	114 (68.3)	2.11 ± .77	.402
		CA	18 (10.8)	2.10 ± .64	
		AA	35 (21.0)	2.28 ± .66	

a Adjusted for gender, age, regions, and preexisting (Day 0) antibody titer.

### 3.3 Association Between Haplotypes and Responsiveness to Influenza Vaccine

As shown in Table 7, we found one haplotype that was related to variation in responsiveness to influenza vaccine. The haplotype consisting of rs41542812—rs17885382—rs2068205—rs41547618—rs6905837—rs9270299—CCTGCA was significantly associated with non-responsiveness to the vaccine compared with GTCATC (OR = 2.39, 95% CI = 1.02–5.62). SNPs were not in LD according to Supplementary Table S3.

**TABLE 7:** Haplotype associations.

HLA haplotype	SNP^a^						Frequency			OR (95%CI)^b^	*P* ^b^
	1	2	3	4	5	6	Total	NR	R		
1	C	C	C	G	C	C	.27	.24	.29	1.00	—
2	C	C	C	A	C	C	.11	.12	.11	1.33 (.87–2.04)	.190
3	C	C	C	G	C	A	.11	.12	.10	1.25 (.82–1.91)	.300
4	C	T	C	G	C	C	.09	.10	.09	1.18 (.76–1.84)	.460
5	C	C	C	G	T	C	.08	.08	.08	1.16 (.69–1.94)	.580
6	C	C	T	G	C	C	.06	.07	.07	1.42 (.82–2.45)	.210
7	G	C	C	G	C	C	.05	.03	.06	0.52 (.25–1.06)	.073
8	C	C	C	A	C	A	.04	.03	.05	0.83 (.42–1.65)	.590
9	C	C	T	G	C	A	.02	.04	.02	2.39 (1.02–5.62)	.046
10	C	C	C	A	T	C	.02	.02	.02	1.32 (.49–3.51)	.580
11	C	T	C	G	T	C	.02	.03	.01	2.16 (.93–5.05)	.075
12	G	C	C	A	C	C	.02	.02	.02	1.51 (.58–3.92)	.400
13	G	C	C	G	C	A	.02	.02	.02	1.34 (.53–3.38)	.530
14	G	C	C	A	C	A	.02	.02	.02	1.14 (.41–3.16)	.810
15	C	T	C	A	C	C	.01	.02	.01	1.70 (.56–5.17)	.350
16	C	T	T	G	C	C	.01	.01	.01	.80 (.20–3.21)	.750
17	G	C	C	G	T	C	.01	.01	.01	2.53 (.61–10.45)	.200
Rare^c^	—	—	—	—	—	—	—	—	—	1.44 (.57–3.61)	.440

a Sequence of SNPs is as follows: 1-rs41542812, 2-rs17885382, 3-rs2068205, 4-rs41547618, 5-rs6905837, 6-rs9270299.

b Adjusted for age and gender logistic regression models. Haplotypes containing SNPs that are associated with responsiveness to influenza vaccine are shown in bold.

c Haplotypes with frequencies <.01.

## 4 Discussion

To further understand variability in HAI antibody response to influenza vaccine, we selected six SNPs in the HLA region to analyze their association with responsiveness and strain-specific antibody titers against the influenza vaccine antigen. According to the results, the effects of HLA SNPs on HAI antibody response to influenza vaccine varied with different vaccine antigens. The most significant results were that rs41547618 was correlated with A/H1N1-specific antibody titer, and rs17885382 was correlated with A/H3N2-specific antibody titer. In this study, we found no HLA SNP with significantly different frequency distribution between responder and non-responder groups, but one haplotype correlated with non-responsiveness to influenza vaccine.

Consistent with the results of previous studies, this study demonstrates that variants in the HLA region were significantly associated with strain-specific antibody titers. Our results showed that the antibody titer of A/H1N1 was low in carriers of the AA genotype at rs41547618 compared to carriers of the GG + GA genotype. According to analysis from Zhou F et al., tag SNP rs41547618 A was in linkage disequilibrium with the HLA-A*1101 allele (R^2 = .894) (Zhou et al., 2016). Poland et al. found that HLA-A*1101 carriers have the higher antibody response to A/H1N1 in Caucasians, which is inconsistent with the results of this study. At the same time, the B/Yamagata-specific antibody titer in the GA genotype carriers at this SNP is higher, suggesting that carriers of the heterozygous genotype may be more conducive to response against influenza vaccine. Given that rs41547618 is located in introns of HLA-A, which encodes HLA class I molecules that mostly present endogenous antigen and activate the cytotoxic effect of CD8^+^ T cells, it seems to be surprised that the variant in this gene was associated with the change of antibody response to influenza vaccine. Nevertheless, there was also evidence discovering that the major histocompatibility complex (MHC) class I molecules could present the exogenous antigen internalized by phagolysosomes (Guermonprez et al., 2003; Houde et al., 2003). This mechanism is called cross-presentation, which might explain the association between rs41547618 and the varied antibody response against the A/H1N1 vaccine antigen. However, the direction and mechanism of this association need to be further studied.

Besides, we also found that the TT genotype of rs17885382 was related to the low level of A/H3N2 antibody compared to the CC + CT genotype. Based on analysis from Zhou F et al., tag SNP rs17885382 T was in linkage disequilibrium with the HLA-DRB1*07:01 allele (*R* ^2^ = 1.000) (Zhou et al., 2016); Gelder et al. found that HLA-DRB1 * 07 allele related to low response of influenza vaccine (Gelder et al., 2002), which is consistent with our results. In addition, the allele is also considered to be related to other immune-mediated phenotypes, such as atopic dermatitis (Weidinger et al., 2013), cockroach allergy (Kalpaklioğlu et al., 2003), and rheumatic heart disease (Rehman et al., 2007). Rs17885382 is in exon 2 of the HLA-DRB1 gene, which encodes the antigen recognition site (Castro-Santos et al., 2020). According to SNPinfo, the T allele is a missense mutation leading to the change of binding pocket of the HLA protein, thus affecting the interaction between influenza A/H3N2 vaccine antigen and the HLA-DRB1 protein. There was little publication about rs17885382 but only one described its correlation with a higher risk of developing asparaginase hypersensitivity (Fernandez et al., 2015). Combined with the results of this study, it is worthy to verify and explore the underlying mechanism of the association between rs17885382 and antibody response to the influenza vaccine using the animal model in future studies.

Although we found no significant SNP with different frequency between responder and non-responder groups, one haplotype consisting of rs41542812—rs17885382—rs2068205—rs41547618—rs6905837—rs9270299—CCTGCA was correlated with non-responsiveness to influenza vaccine compared with GTCATC. These results may infer that vaccine response was a complex process involving multiple factors. The effect of haplotype may be added up by the tiny influence of each SNP, thus reaching a statistically significant level. Apart from providing a better understanding of the varied immune response to influenza vaccine in the population, the effect of SNPs and haplotypes discovered in this study may also help optimize the vaccine design by refining peptide filtering and scoring strategies for vaccine candidate selection (Liu et al., 2020). Therefore, peptides with better binding affinity to the HLA pocket area, which overcome the negative effect of variants found by this study, should be considered when designing influenza vaccine with improved immunogenicity.

However, our study still has several limitations. First, the HAI antibody measured by this study only captured humoral antibody response to the HA protein of the vaccine antigen. Antibodies stimulated by other vaccine components such as neuraminidase may provide more insights into the effects of HLA SNPs on humoral and cellular immune responses induced by influenza vaccines. More comprehensive indicators should be examined in the future. Second, subjects with influenza vaccine history were excluded in this study, but we still could not eliminate the effect of prior immunity gained by infection. Third, this study only included Han nationality as study subjects. Although we compared our findings with similar studies conducted in other populations, a stratified analysis within the same study still is the best way to compare the effect between different populations. Finally, other immune-related genes and health conditions such as obesity and chronic diseases, which cannot be analyzed and discussed in this article, may also affect the HAI antibody response to influenza vaccine.

To the best of our knowledge, this is the first study to examine the association between HLA SNPs and HAI antibody response to influenza vaccine in the Han nationality, which can help in a better understanding of the varied antibody response in influenza vaccine recipients and optimize the vaccine design strategy. The underlying mechanism of HLA SNPs’ effect on HAI antibody response to influenza vaccine needs to be further verified.

## Data Availability

The raw data that support the findings of this study are available from the corresponding author, upon reasonable request.
