# Supplementary material for: Single Nucleotide Polymorphisms in the Human Leukocyte Antigen Region Are Associated With Hemagglutination Inhibition Antibody Response to Influenza Vaccine
Source: Front Genet. 2022 Feb 7;13:790914. doi: 10.3389/fgene.2022.790914 (PMC8859407; doi:10.3389/fgene.2022.790914)
Supplement: Supplementary file 1 [file DataSheet1.docx]

Supplementary Material

# Supplementary Tables

**Supplementary Table 1.** Vaccine strains of TIV administrated in this study from 2009-2019.

| Season | A/H1N1 | A/H3N2 | B/Victoria or B/Yamagata |
| --- | --- | --- | --- |
| 2009-2010 | A/Brisbane/59/2007 (H1N1) | A/Brisbane/10/2007 (H3N2) | B/Brisbane/60/2008(Victoria) |
| 2010-2011 | A/California/7/2009 (H1N1) | A/Perth/16/2009 (H3N2) | B/Brisbane/60/2008(Victoria) |
| 2011-2012 | A/California/7/2009 (H1N1) | A/Perth/16/2009 (H3N2) | B/Brisbane/60/2008(Victoria) |
| 2012-2013 | A/California/7/2009 (H1N1)pdm09 | A/Victoria/361/2011 (H3N2) | B/Wisconsin/1/2010(Yamagata) |
| 2013-2014 | A/California/7/2009 (H1N1)pdm09 | A/Victoria/361/2011 (H3N2) | B/Massachusetts/2/2012(Yamagata) |
| 2014-2015 | A/California/7/2009 (H1N1)pdm09 | A/Texas/50/2012 (H3N2) | B/Massachusetts/2/2012(Yamagata) |
| 2015-2016 | A/California/7/2009 (H1N1)pdm09 | A/Switzerland/9715293/2013 (H3N2) | B/Phuket/3073/2013(Yamagata) |
| 2016-2017 | A/California/7/2009 (H1N1)pdm09 | A/Hong Kong/4801/2014 (H3N2) | B/Brisbane/60/2008(Victoria) |
| 2017-2018 | A/Michigan/45/2015 (H1N1)pdm09 | A/Hong Kong/4801/2014 (H3N2) | B/Brisbane/60/2008(Victoria) |
| 2018-2019 | A/Michigan/45/2015 (H1N1)pdm09 | A/Singapore/INFIMH-16-0019/2016 (H3N2) | B/Colorado/06/2017(Victoria) |

**Supplementary Table 2.** Selection and detection rate of HLA tag SNPs

| Author [Ref] | Allele | Tag SNPs | Tag SNPs allele | MAFs | R^2^ | Call rate (%) |
| --- | --- | --- | --- | --- | --- | --- |
| (Gelder et al., 2002) | HLA-DRB1*07 | rs17885382 | T | 0.091 | 1.000 | 95.6 |
| (Gelder et al., 2002) | HLA-DQB1*0303 | rs6905837 | T | 0.160 | 0.925 | 96.5 |
| (Poland et al., 2008) | HLA-A*1101 | rs41547618 | A | 0.190 | 0.894 | 98.1 |
| (Poland et al., 2008) | HLA-DQB1*0502 | rs41542812 | G | 0.060 | 1.000 | 98.6 |
| (Narwaney et al., 2013) | HLA-DRB1*15 | rs9270299 | A | 0.131 | 1.000 | 95.9 |
| (Moss et al., 2013) | HLA-DPB1*0401 | rs2068205 | T | 0.110 | 0.872 | 98.6 |

# Supplementary Table 3. The linkage disequilibrium coefficient among six SNPs in HLA.

|  | rs41542812 | rs17885382 | rs2068205 | rs41547618 | rs6905837 | rs9270299 |
| --- | --- | --- | --- | --- | --- | --- |
| rs41542812 | - | 0.8891 | 0.4679 | 0.1312 | 0.4024 | 0.1689 |
| rs17885382 | 0.0168 | - | 0.3261 | 0.5453 | 0.0098 | 0.9980 |
| rs2068205 | 0.0041 | 0.0023 | - | 0.4461 | 0.7061 | 0.0572 |
| rs41547618 | 0.0071 | 0.0154 | 0.0100 | - | 0.2304 | 0.0576 |
| rs6905837 | 0.0037 | 0.0001 | 0.0117 | 0.0030 | - | 0.9621 |
| rs9270299 | 0.0142 | 0.0426 | 0.0017 | 0.0027 | 0.0428 | - |

# Values on the left of “-” are r^2^ and those on the right are Lewontin’s D’ coefficients.

# Reference

Gelder, C. M., Lambkin, R., Hart, K. W., Fleming, D., Williams, O. M., Bunce, M., et al. (2002). Associations between human leukocyte antigens and nonresponsiveness to influenza vaccine. *J Infect Dis* 185(1), 114-117. doi: 10.1086/338014.

Moss, A. J., Gaughran, F. P., Karasu, A., Gilbert, A. S., Mann, A. J., Gelder, C. M., et al. (2013). Correlation between human leukocyte antigen class II alleles and HAI titers detected post-influenza vaccination. *PLoS One* 8(8), e71376. doi: 10.1371/journal.pone.0071376.

Narwaney, K. J., Glanz, J. M., Norris, J. M., Fingerlin, T. E., Hokanson, J. E., Rewers, M., et al. (2013). Association of HLA class II genes with clinical hyporesponsiveness to trivalent inactivated influenza vaccine in children. *Vaccine* 31(7), 1123-1128. doi: 10.1016/j.vaccine.2012.12.026.

Poland, G. A., Ovsyannikova, I. G., and Jacobson, R. M. (2008). Immunogenetics of seasonal influenza vaccine response. *Vaccine* 26, D35-D40. doi: 10.1016/j.vaccine.2008.07.065.
